# Rac1 is a downstream effector of PKCα in structural synaptic plasticity

**DOI:** 10.1038/s41598-020-58610-6

**Published:** 2020-02-04

**Authors:** Xun Tu, Ryohei Yasuda, Lesley A. Colgan

**Affiliations:** 1 Neuronal Signal Transduction Group, Max Planck Florida Institute for Neuroscience, Jupiter, FL USA; 2 International Max Planck Research School for Brain and Behavior, Jupiter, FL USA; 3 FAU/Max Planck Florida Institute Joint Graduate Program in Integrative Biology and Neuroscience, Florida Atlantic University, Boca Raton, FL USA

**Keywords:** Molecular neuroscience, Spine plasticity, Long-term potentiation

## Abstract

Structural and functional plasticity of dendritic spines is the basis of animal learning. The rapid remodeling of actin cytoskeleton is associated with spine enlargement and shrinkage, which are essential for structural plasticity. The calcium-dependent protein kinase C isoform, PKCα, has been suggested to be critical for this actin-dependent plasticity. However, mechanisms linking PKCα and structural plasticity of spines are unknown. Here, we examine the spatiotemporal activation of actin regulators, including small GTPases Rac1, Cdc42 and Ras, in the presence or absence of PKCα during single-spine structural plasticity. Removal of PKCα expression in the postsynapse attenuated Rac1 activation during structural plasticity without affecting Ras or Cdc42 activity. Moreover, disruption of a PDZ binding domain within PKCα led to impaired Rac1 activation and deficits in structural spine remodeling. These results demonstrate that PKCα positively regulates the activation of Rac1 during structural plasticity.

## Introduction

Dendritic spines of pyramidal neurons in the hippocampus undergo activity-dependent structural and functional plasticity that has been reported to be crucial for learning and memory^1–3^. This plasticity is mediated by the coordinated regulation of complex signaling networks that transduce short-lived synaptic input into long-lasting biochemical changes to modulate the strength and structure of synapses and, ultimately, animal behavior^4,5^.

Protein kinase C (PKC) is a family of serine/threonine kinases that have long been implicated as essential for synaptic plasticity, learning and memory. Inhibition of PKC blocks LTP induction and also disrupts the maintenance of pre-established LTP^6,7^. In addition, pharmacologically activating PKC or overexpressing constitutively active PKC potentiates synapses and enhances learning^8,9^. PKC exists as at least 10 isozymes that are categorized into three subfamilies: the classic isozymes (PKCα, PKCβ and PKCγ), the novel isozymes (PKCδ, PKCɛ, PKCη, and PKCθ) and the atypical isozymes (PKCζ and PKCλ/ι). The classic PKC isozymes, ubiquitously expressed in the brain, transduce signals dependent on Ca^2+^ and diacylglycerol (DAG)^10–13^. Recently, PKCα was demonstrated to be uniquely required for structural LTP in hippocampal dendritic spines. This specificity was defined by a four amino acid ﻿C-terminal PDZ-binding motif (QSAV)^14^. Evidence suggests that PKCα activity integrates neurotrophic signaling, including the activation of TrkB, with Ca^2+^ influx through NMDARs to facilitate the induction the plasticity in dendritic spines^14^. However, the downstream molecular mechanisms through which PKCα facilitates structural synaptic plasticity remain unknown.

The expression of structural plasticity, through spine enlargement and insertion of additional glutamate receptors, requires actin remodeling through the regulated activity of small GTPases including Rac1, Cdc42 and Ras^15–19^. These small GTPases are precisely coordinated across spatiotemporal domains by a complex network of GTPase accelerating proteins (GAPs) and GTPase exchange factors (GEFs), which regulate GTPase activity to induce cytoskeletal remodeling ﻿crucial for activity-dependent spine plasticity^1,20,21^. PKC has been linked to actin regulation in many cell types. In neurons, application of phorbol esters, which activate PKC, can induce structural changes including growth cone retraction or collapse and the formation of lamellae in dendrites^22,23^. These data suggest that PKC can regulate actin through modulating small GTPase function, however whether these pathways are involved during structural plasticity of spines and which small GTPases might be regulated are unknown^24^.

## Results

Here, we examine whether PKCα regulates small GTPases during the induction of plasticity.

**Figure 1:**
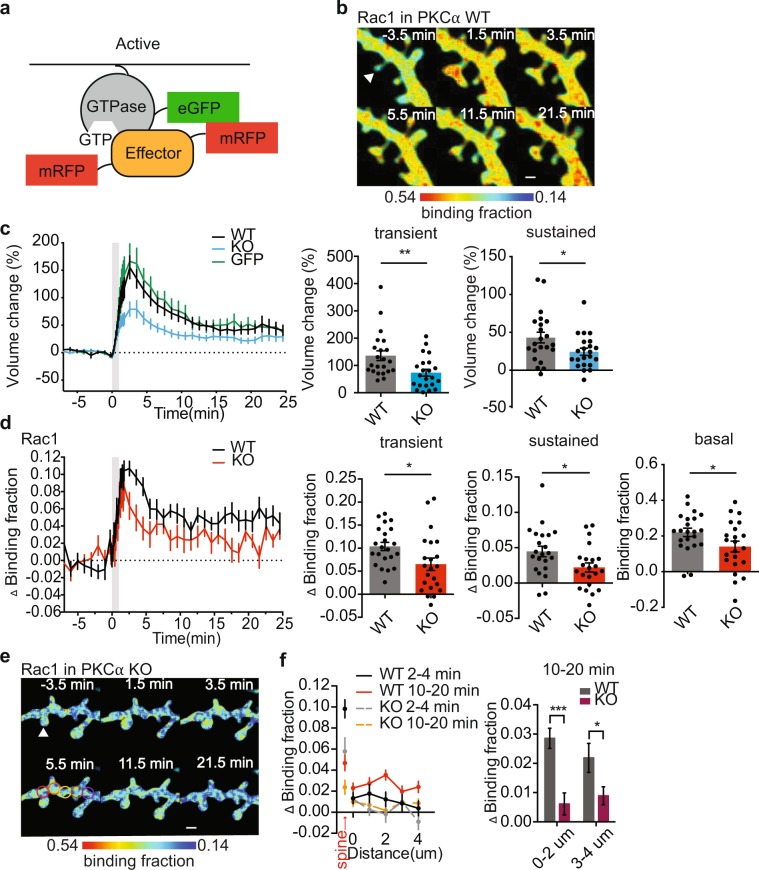
PKC regulates Rac1 activation during sLTP. (**a**) Schematic of small GTPase FRET sensors. (**b**) 2pFLIM images of Rac1 activation in WT slices at indicated time points. Arrowhead represents point of uncaging. Warmer colors indicate higher binding fraction of sensor and higher Rac1 activity. Scale bar, 1 μm. (**c**) Time courses and quantification of transient (1–3 min) and sustained (10–25 min) spine volume change induced by glutamate uncaging in neurons expressing GFP or in neurons from PKCα WT and KO littermates expressing Rac1 sensor. (**d**) Time courses and quantification of transient (1.5–3.5 min), sustained (10–25 min) and basal (−8–0 min) Rac1 activation in stimulated spines from WT and PKCα KO littermates. (**e**) 2pFLIM images of Rac1 activation in PKCα KO slices at indicated time points. Arrowhead represents point of uncaging. Spreading of Rac1 activation in the dendrite were measured in the regions 0 μm from the stimulated spine (red), 1 μm (orange), 2 μm (yellow), 3 μm (brown) and 4 μm (purple). Scale bar, 1 μm. (**f**) Spatial profile and quantification of spreading Rac1 activation along the dendrite at indicated times and distances from the stimulated spine in WT and PKCα KO littermates. Data are mean ± s.e.m. Grey shading indicates time of uncaging. *P < 0.05, **P < 0.01 two-tailed t-test (**c,d**) and two-way ANOVA with Sidak’s mutiple comparisons test (**f**). n (neurons/spines) = 18/22 WT, 19/22 PKCα KO and 5/11 GFP.

In order to study signaling in single spines during structural plasticity, we combined two-photon release of caged glutamate^25^, fluorescence resonance energy transfer (FRET)-based sensors, and two-photon fluorescence lifetime imaging microscopy (2pFLIM)^26^ to monitor the dynamics of intracellular signaling events with high spatiotemporal resolution. Specifically, using previously published FRET sensors, we monitored the spatiotemporal activation of the actin-regulating small GTPases, including Ras^27–29^, Cdc42^15^ and Rac1^19^ during the induction of structural long-term potentiation (sLTP). ﻿Briefly, these sensors are composed of ﻿two components: (1) full length GTPase fused to green fluorescent protein (GTPase–eGFP), and (2) a specific GTPase binding domain of a downstream effector fused to two copies of red fluorescent protein (mRFP-effector-mRFP) (Fig. 1a). Activation of the GTPase increases the affinity between the two components of the sensor and the FRET between the fluorophores. Using 2pFLIM we can measure small GTPase activation by monitoring changes in the binding of the sensor components (binding fraction) through  quantitatively measuring decreases in the fluorescence lifetime of GFP.

We transfected organotypic hippocampal slices from wildtype (WT) or PKCα knockout (KO) mice with small GTPase sensors and imaged CA1 pyramidal neurons using 2pFLIM. ﻿In response to glutamate uncaging targeted to a single dendritic spine (30 pulses at 0.5 Hz), the stimulated spine from WT slices rapidly enlarged by ~150% (transient phase) and persisted with an increased volume of ~40% (sustained phase) lasting at least 25 min (Fig. 1b,c). This sustained increase in the spine volume is highly correlated with increases in the functional strength of the stimulated spine^28,30^. Consistent with previous work, ﻿hippocampal CA1 neurons from PKCα KO mice ﻿showed a deficit in sLTP^14^. Moreover, sLTP of stimulated spines expressing Rac1 sensor was consistent with spines expressing eGFP alone (Fig. 1c), demonstrating, and consistent with previous characterization^15,19^, that effects of sensor overexpression on endogenous signaling are minimal. ﻿When sLTP was induced in single dendritic spines, we observed rapid and sustained activation of Rac1 (Fig. 1b,d) in the WT slices that was consistent with previously reported findings^19^. However, we found that in the absence of PKCα, Rac1 activity is lower at baseline and its activation is significantly attenuated during sLTP (Fig. 1d,e). Rac1 activity in WT mice was restricted to the stimulated spines at early time points (2–4 min), but spread into the dendrite and nearby spines at later time points (10 – 20 min), consistent with a previous study^19^. In PKCα KO mice, Rac1 activity in both stimulated spines and adjacent dendrites were attenuated (Fig. 1e,f). Thus, PKCα positively regulates Rac1 activation to facilitate plasticity.

**Figure 2:**
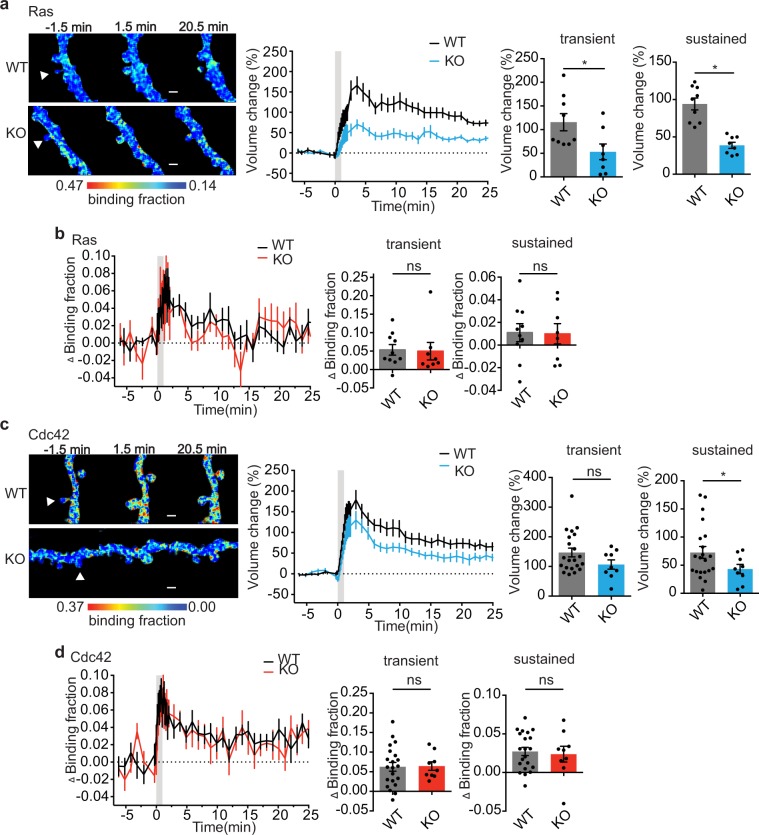
PKCα does not regulate Ras or Cdc42 activation during sLTP. (**a,c**) 2pFLIM images of Ras (**a**) and Cdc42 (**c**) activation in PKCα WT and KO neurons at indicated time points. Time courses and quantification of transient (1–3 min) and sustained (10–25 min) spine volume change induced by glutamate uncaging in neurons expressing Ras1 sensor (**a**) or Cdc42 sensor (**c**) from PKCα WT and KO littermates. (**b,d**) Time courses and quantification of transient (1–3 min) and sustained (10–25 min) Ras1 (**b**) or Cdc42 (**d**) activation in stimulated spines from WT and PKCα KO littermates. Data are mean ± s.e.m. n(neurons/spines) Ras: n = 8/10 WT, n = 7/8 KO. Cdc42: n = 15/21 WT and n = 7/9 KO *P < 0.05, two-tailed t-test.

In order to test the specificity of PKCα signaling toward Rac1, we tested whether the plasticity-induced activation of other small GTPases involved in plasticity, Ras or Cdc42^1,31,32^, was also impaired in the absence of PKCα. Impairment of the functionally relevant sustained phase of sLTP was observed in PKCα KO neurons transfected with Ras or Cdc42 sensor (Fig. 2a,c). However, the activation of these two small GTPases during sLTP were not affected by loss of PKCα (Fig. 2b,d). This demonstrates that Rac1 but not Ras or Cdc42 is downstream of PKCα during sLTP.

**Figure 3:**
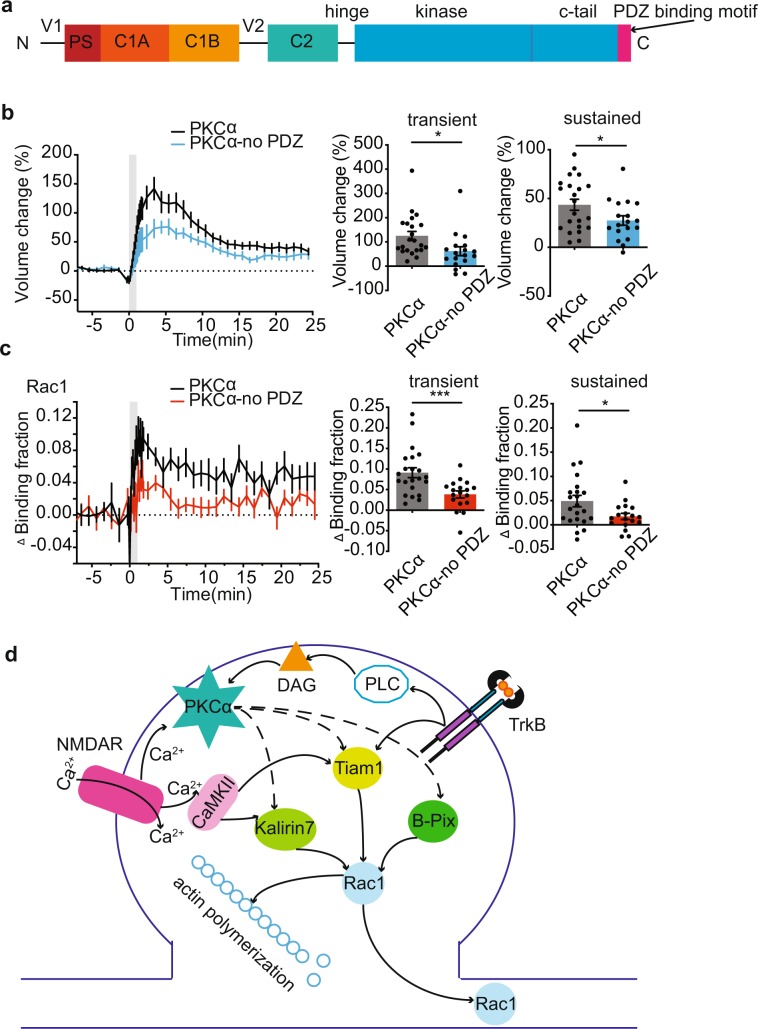
PKCα regulates Rac1 activation during sLTP via PDZ binding domain. (**a**) Primary structure of PKCα showing pseudosubstrate, C1A and C1B domains, C2 domain, kinase domain, C-terminal tail, and PDZ binding motif. (**b,c)** Time courses and quantification of transient (1–3 min) and sustained (10–25 min) spine volume change **(b)** and transient and sustained Rac1 activation **(c)** induced by glutamate uncaging in PKCα KO hippocampal neurons expressing PKCα or PKCα without PDZ domain (PKCα-no PDZ). n = 20/22 PKCα and 14/18 PKCα-no PDZ (neurons/spines). Data are mean ± s.e.m. *P < 0.05, two-tailed t-test. (**d**) Schematic of potential PKCα regulation of Rac1.

We next investigated whether the PDZ binding domain of PKCα, which was shown to be critical for its isozyme-specific signaling in spine plasticity^14^, was required for downstream regulation of Rac1 (Fig. 3a). PKCα or PKCα lacking its PDZ binding domain was sparsely and postsynaptically expressed alongside the Rac1 sensor in hippocampal slices from PKCα KO mice. We found impairment of sLTP (Fig. 3b) and Rac1 activation (Fig. 3c) only when the PDZ binding domain was disrupted. This finding suggests a crucial role for PDZ binding domain of PKCα in Rac1 activation during sLTP.

## Discussion

In this study, we have described that PKCα regulates the activation of Rac1, but not Ras or Cdc42, during sLTP of dendritic spines (Fig. 3d). This modulation relies on PKCα’s PDZ-binding motif, which may localize PKCα to a signaling complex scaffold that also recruits Rac1 GTPase-activating proteins (GAPs) or Guanine nucleotide-exchange factors (GEFs). Although the specific mechanism through which PKCα regulates Rac1 activity remains to be determined, potential scaffolds include PICK1, SAP-97 or PSD-95, which can interact with PKCα through its C-terminal PDZ binding domain and have been implicated in plasticity of spines^33–38^.

The regulation of Rac1 during plasticity through GAPs and GEFs is highly complex in order to allow for tightly-regulated remodeling of various cytoskeletal domains in a precise spatial and temporal pattern^39^. Rac1 has been shown to be localized to signaling complexes that scaffold upstream activators, small GTPases and downstream effectors, enabling subcompartmentalization of functional output^40^. Rac1 activation promotes F-actin polymerization that facilitates spine growth and stabilization through the activation of major downstream targets p21-activated kinase (PAK), LIM kinase I (LIMKI) and WAVE regulatory complex (WRC)^31,41^. The phosphorylation of PAK and LIMKI leads to inactivation of actin depolymerizing factor ADF/cofilin, which in turn stimulates F-actin polymerization^1,31^. Additionally, Rac1 regulates actin branching through activation of WRC which drives Arp2/3^41,42^. Interestingly, PICK1 has also been shown to bind and regulate Arp2/3^43^.

While PKCα could modulate Rac1 through inhibition of a GAP or Rho GDP dissociation inhibitors (RhoGDIs)^44–46^, much of the specificity of Rac1 GTPase signaling is reportedly regulated by GEFs^46–48^. At least ten different RhoGEFs have been identified to be localized to the post-synaptic density^49^. Of these, Kalirin 7, Tiam1 and B-Pix all show preferential GEF activity for Rac1, are able to be phosphorylated by classic PKC isozymes *in-vitro* and are required for sLTP of spines^50–57^. Tiam1 contains a PDZ domain that could potentially interact with PKCα, however the structure of its PDZ domain does not predict PKCα as a preferred binding partner^58^. Kalirin7 and B-Pix also each contain a PDZ binding domain, which could localize them to PDZ containing-scaffolds together with activated PKCα^57,59,60^. Indeed Kalirin-7 binds scaffolds including PSD-95, PICK1 and SAP97 *in vitro*. Only its interaction with PSD-95 has been confirmed in hippocampal neurons^61^.

It is important to note that these experiments rely on overexpression of small GTPase sensors. Importantly the effects of sensor overexpression on volume change of stimulated spines, sensor activation and kinetics have been well characterized and show minimal effects in WT neurons^15,19,28^. However, as observed in Fig. 2c, PKCα KO animals did not have significantly impaired transient plasticity. Thus, it is possible that overexpression of Cdc42 may partially rescue the impairment of plasticity in a KO background. This finding is interesting given the known crosstalk between Cdc42 and Rac1 in regulating actin dynamics^62^. Rac1 and Cdc42 share several common effectors^63^ that are relevant to structural plasticity of spines. For example, both activated Rac and Cdc42 can increase PAK1 association with LIMK and LIMK-dependent phosphorylation of cofilin^64^. Cofilin phosphorylation has been shown to be an important regulator of actin remodeling during structural plasticity of spines and is transiently increased during the early phase (~2 min) of plasticity^65^. Thus, overexpression of Cdc42 may alleviate some of the PKCα – Rac1 mediated KO deficits. Further study on potential crosstalk between Cdc42 and Rac1, particularly related to cofilin regulation during structural plasticity of spines will be informative.

Both Rac1 and PKCα activation are downstream of TrkB receptor activation in the stimulated spine^14,19^ (Fig. 3d). One important future direction is to determine if TrkB-dependent activation of Rac1 is solely through PKCα or whether a more complex feedback loop is in play. The increased activity and spreading of Rac1 promoted by ﻿PKCα ﻿is consistent with an essential role for PKCα in integrating neurotrophic signals to facilitate plasticity. Moreover, this link between PKCα and Rac1 supports a growing understanding of the molecular mechanisms underlying ﻿heterosynaptic facilitation, whereby the induction of plasticity in one spine lowers the threshold of plasticity induction in nearby spines^14,28,66^.

This study identified a novel molecular pathway that links short-lived calcium influx and activation of PKCα, to long-lasting Rac1 activation and changes in spine structure. This work, taken together with previous findings that another Ca^2+^-dependent kinase, CaMKII, also activates Rac1 during sLTP^57,66^, Rac1 appears to be a key convergence point of multiple upstream calcium-dependent pathways (Fig. 3d). We anticipate that the better understanding of Rac1 signaling pathway may ultimately help us to uncover the signaling network with which memories are encoded.

## Materials and Methods

### Animals

All experimental procedures were approved and carried out in accordance to the regulations of the Max Planck Florida Institute for Neuroscience Animal Care and Use Committee in accordance with guidelines by the US National Institutes of Health. P4-P8 mouse pups from both sexes were used for organotypic slices for imaging studies. The genotype of each animal was verified before preparing slices. PKCα KO 129/sv animals were received from Dr. Michael Leitges. Animals were crossed to C57Bl/6N Crl and are on a mixed background. For all the experiments, WT littermates were used as controls for KO animals.

### Plasmids

All the plasmids used were previously developed and described in publication and are available on Addgene^14,19,67^. Briefly, the Rac1 sensor consisted of mEGFP-Rac1 (Addgene #83950) and mCherry-PAK2 binding domain-mCherry (Addgene #83951), the Ras sensor consisted of pCI-mEGFP-HRas ((Addgene #18666) and ﻿pCI-mRFP-RBD^K65E,K108A^- mRFP (Addgene #45149), the Cdc42 sensor consisted of mEGFP-Cdc42 (Addgene #29673) and mCherry-Pak3(60-113)/S74A/F84A-mCherry-C1 (Addgene #29676). PKCα (*Bos taurus*) was cloned into CMV-promoter-containing mEGFP C1 vectors such that mEGFP was fused to the N terminus of PKCα^14^. mEGFP-PKCα∆QSAV was made by introducing a single point mutation into mEGFP-PKCα/C1 to introduce a stop codon before the last four amino acids (QSAV) of PKCα^14^.

### Organotypic hippocampal slice cultures and transfection

Organotypic hippocampal slices were prepared from wildtype or transgenic postnatal 4–8 day old mouse pups of both sexes as previously described^68^. In brief, the animals were anaesthetized with isoflurane, after which the animal was quickly decapitated and the brain removed. The hippocampi were dissected and cut into 350 µm thick coronal hippocampal slices using a McIlwain tissue chopper (Ted Pella, Inc) and plated on hydrophilic PTFE membranes (Millicell, Millipore) fed by culture medium containing MEM medium (Life Technologies), 20% horse serum, 1mM L-Glutamine, 1 mM CaCl_2_, 2 mM MgSO_4_, 12.9 mM D-Glucose, 5.2 mM NaHCO_3_, 30 mM Hepes, 0.075% Ascorbic Acid, 1 µg/ml Insulin. Slices were incubated at 37 °C in 5% CO^2^. After 7–12 days in culture, CA1 pyramidal neurons were transfected with biolistic gene transfer^69^ using 1.0 µm gold beads (8–12 mg) coated with plasmids containing 50 μg of total cDNA of interest in the following ratios. ﻿Rac1 sensor, donor: acceptor = 1:2; Rac1 sensor plus PKCα, donor: acceptor: PKCα = 1:2:1; Rac1 sensor plus PKCα without PDZ binding domain, donor: acceptor: PKCα- no PDZ domain = 1:2:1; Ras sensor, donor: acceptor = 1:3; Cdc42 sensor, donor: acceptor = 1:1). Neurons expressing all plasmid combinations were imaged 2–5 days after transfection.

### 2pFLIM

FLIM imaging using a custom-built two-photon fluorescence lifetime imaging microscope was performed as previously described^15^. 2pFLIM imaging was performed using a Ti-sapphire laser (Coherent, Cameleon) at a wavelength of 920 nm with a power of 1.4–1.6 mW. Fluorescence emission was collected using an immersion objective (60×, numerical aperture 0.9, Olympus), divided with a dichroic mirror (565 nm) and detected with two separated photoelectron multiplier tubes placed after wavelength filters (Chroma, 510/70-2p for green and 620/90-2p for red). Both red and green channels were fit with photoelectron multiplier tubes (PMT) having a low transfer time spread (H7422-40p; Hamamatsu) to allow for fluorescence lifetime imaging. Photon counting for fluorescence lifetime imaging was performed using a time-correlated single photon counting board (SPC-150; Becker and Hickl) and fluorescence images were acquired with PCI-6110 (National instrument) using modified ScanImage^70^(https://github.com/ryoheiyasuda/FLIMimage_Matlab_ScanImage). Intensity images for analysis of sLTP volume changes were collected by 128 × 128 pixels as a z stack of three slices with 1 µm separation and averaging 6 frames/slice. Spine volume was measured as the integrated fluorescent intensity of EGFP after subtracting background (F). Spine volume change was calculated by F/F_0_, in which F_0_ is the average spine intensity before stimulation.

### Two-photon glutamate uncaging

A second Ti-sapphire laser tuned at a wavelength of 720 nm was used to uncage 4-methoxy-7-nitroindolinyl-caged-l- glutamate (MNI-caged glutamate) in extracellular solution with a train of 4–8 ms, 2.8–3.0 mW pulses (30 times at 0.5 Hz) in a small region ~0.5 µm from the spine of interest as previously described^14^. Experiments were performed in Mg^2+^ fee artificial cerebral spinal fluid (ACSF; 127 mM NaCl, 2.5 mM KCl, 4 mM CaCl_2_, 25 mM NaHCO_3_, 1.25 mM NaH_2_PO_4_ and 25 mM glucose) containing 1 µM tetrodotoxin (TTX) and 4 mM MNI-caged L-glutamate aerated with 95% O_2_ and 5% CO_2._ Experiments were performed at room temperature.

### 2pFLIM analysis

To measure the fraction of donor that was undergoing FRET with acceptor (Binding Fraction), we fit a fluorescence lifetime curve summing all pixels over a whole image with a double exponential function convolved with the Gaussian pulse response function:

$$F(t)={F}_{0}[{P}_{D}H(t,{t}_{0},\,{\tau }_{D},\,{\tau }_{G})+{P}_{AD}H(t,{t}_{0},\,{\tau }_{AD},\,{\tau }_{G})]$$

where τ_AD_ is the fluorescence lifetime of donor bound with acceptor, *P*_D_ and *P*_AD_ are the fraction of free donor and donor undergoing FRET with acceptor, respectively, and *H*(*t*) is a fluorescence lifetime curve with a single exponential function convolved with the Gaussian pulse response function:

$$H(t,\,{t}_{0},\,{t}_{D},\,{t}_{G})=\frac{1}{2}\exp (\frac{{\tau }_{G}^{2}}{2{\tau }_{D}^{2}}-\frac{t-{t}_{0}}{{\tau }_{i}}){\rm{erfc}}(\frac{{\tau }_{G}^{2}-{\tau }_{D}(t-{t}_{0})}{\surd 2{\tau }_{D}{\tau }_{G}}),$$

in which τ_D_ is the fluorescence lifetime of the free donor, τ_G_ is the width of the Gaussian pulse response function, *F*_0_ is the peak fluorescence before convolution and *t*_0_ is the time offset, and erfc is the complementary error function.

We fixed τ_D_ to the fluorescence lifetime obtained from free eGFP (2.6 ns), and then fixed τ_AD_ to fluorescence lifetime of the donor bound with acceptor (1.1 ns). For experimental data, we fixed τ_D_ and τ_AD_ to these values to obtain stable fitting.

To generate the fluorescence lifetime image, we calculated the mean photon arrival time, <*t*>, in each pixel as:

$$ < {t} > =\int \,{t}F({t})dt/\int \,{F}({t}){dt},$$

Then, the mean photon arrival time is related to the mean fluorescence lifetime, <*τ*>, by an offset arrival time, *t*_*o*_, which is obtained by fitting the whole image:

$$ < {T} > = < {t} > -{{t}}_{0}.$$

For small regions-of-interest (ROIs) in an image (spines or dendrites), we calculated the binding fraction (P_AD_) as:

$${{P}}_{AD}={\tau }_{D}({T}_{D}- < T > ){({T}_{D}-{T}_{AD})}^{-1}{({T}_{D}+{T}_{AD}- < T > )}^{-1}.$$

Data with lifetime fluctuations in the baseline that were greater than 0.15 ns were excluded before further analysis. To analyse the spatial spreading of Rac1 activation, contiguous 1 μm diameter ROIs along dendrite from the base of the stimulated spines were analysed for Rac1 activation over time.

### Statistical analysis

All values are presented as mean ± SEM unless otherwise noted. Number of independent measurements (n[neurons/spines]) is indicated in figure legends. Unpaired two-tailed student’s t test was used for comparing two independent samples. Two-way ANOVA followed by multiple comparison test was used to compare grouped data sets (Prism 6, GraphPad). Data were excluded if obvious signs of poor cellular health (for example, dendritic blebbing, spine collapse) were apparent. In addition, outlier analysis was performed on spine volume data with ROUT (Q = 1%).This analysis led to the exclusion of 2 out of 113 spines from analysis.
